# Apigetrin Abrogates Lipopolysaccharide-Induced Inflammation in L6 Skeletal Muscle Cells through NF-κB/MAPK Signaling Pathways

**DOI:** 10.3390/cimb44060180

**Published:** 2022-06-08

**Authors:** Sang-Eun Ha, Pritam Bhagwan Bhosale, Hun-Hwan Kim, Min-Yeong Park, Abuyaseer Abusaliya, Gon-Sup Kim, Jin-A Kim

**Affiliations:** 1 Research Institute of Life Science, College of Veterinary Medicine, Gyeongsang National University, Jinju 52828, Korea; sangdis2@naver.com (S.-E.H.); shelake.pritam@gmail.com (P.B.B.); shark159753@naver.com (H.-H.K.); lilie17@daum.net (M.-Y.P.); yaseerbiotech21@gmail.com (A.A.); 2 Department of Physical Therapy, International University of Korea, Jinju 52833, Korea

**Keywords:** apigetrin, L6, anti-inflammation, NF-κB, MAPK

## Abstract

Apigetrin is a glycosidic flavonoid derived from *Teucrium gnaphalodes* that has a wide range of biological activities, including antioxidant, anti-inflammatory, and anticancer. Inflammation is a kind of defense mechanism in the body. Flavonoids are natural phytochemicals that exert anti-inflammatory effects in numerous cells. In the present study, we investigated the anti-inflammatory effect of apigetrin and its underlying mechanism of activity in skeletal muscle cells (L6). The determination of cytotoxicity was performed by MTT assay. We treated L6 cells with apigetrin, and nontoxic concentrations were chosen to perform further experimentation. Apigetrin inhibited the expression of iNOS and COX-2 induced by LPS in a dose-dependent manner. iNOS and COX-2 are inflammatory markers responsible for enhancing the inflammatory response. Apigetrin also inhibited the LPS-induced phosphorylation of p65 and IκB-α. NF-κB signaling regulates the inflammatory process by mediating various proinflammatory genes. Similarly, the MAPK signaling pathway consists of ERK, JNK, and p38, which plays a critical role in the production of cytokines and downstream signaling events leading to inflammation. Apigetrin significantly downregulated the phosphorylation of JNK and p38, but did not affect the phosphorylation of ERK in the LPS-stimulated cells. These findings indicate the correlation between the anti-inflammatory activity of NF-κB and the MAPK signaling pathway. Thus, our overall finding suggests that apigetrin has anti-inflammatory effects and it can be considered for further drug design on L6 skeletal muscle cells.

## 1. Introduction

Apigetrin (4′,5-dihydryxyflavone 7-O-glucoside) is a naturally occurring flavonoid abundantly present in various vegetables, fruits, beans, and tea leaves. Apigetrin has 4′ hydroxyl groups at position seven; it contains a glucoside residue, as well as a C2–C3 double bond, which contributes to its unique physicochemical characteristics [1]. The regulatory mechanisms and immunomodulatory effect of apigetrin have been demonstrated in numerous studies [2,3]. Apigetrin contains high levels of anti-inflammatory, antioxidant, antiviral, antibacterial, antiangiogenic, and mild tumor-suppressive properties. Apigetrin is characterized by immunometabolic changes, which enhance the production of proinflammatory cytokines, thus, suppressing cytokine production in response to inflammation [4,5,6]. From this point of view, it is crucial to use skeletal muscle cells with inflammation as a cellular disease model to determine the underlying mechanism of a potential therapeutic drug.

Inflammation is involved in the pathogenesis of lipopolysaccharide (LPS)-induced damage [7]. LPS has been implicated as a major cause of inflammation and an important component of the cell wall of Gram-negative bacteria [8]. Additionally, it stimulates the production and secretion of cytokines and other inflammatory mediators in skeletal muscle cells, resulting in a proinflammatory response [9,10]. Muscle weakness, muscle mass loss, and poor functionality are all symptoms of chronic inflammation [11]. The phosphorylation of mitogen-activated protein kinases (MAPKs) and the nuclear translocation of nuclear factor-kappa B (NF-κB) occur in response to LPS [12].

NF-κB and MAPK are anti-inflammatory cytokines that curb inflammation and modulate metabolic pathways. Apigetrin can inhibit inflammation through NF-κB and MAPK, thus, indicating that apigetrin may possess a potential protective effect against LPS-induced damage in L6 skeletal muscle cells. The activation of NF-κB signaling is a critical molecular mechanism for inflammatory reactions, as it causes the production of several proinflammatory genes, such as inducible nitric oxide synthase (iNOS), cyclooxygenase-2 (COX-2), and tumor necrosis factor-alpha (TNF-α). The NF-κB family consists of several subunits, including p65 (Rel-A), Rel-B, c-Rel, p50, and p52 [13]. The NF-κB p65 subunit has received substantial attention as a key target protein for inflammation due to the disproportionate increase in the p65-mediated transactivation of proinflammatory mediators [14]. Additionally, signal-induced ubiquitination and the subsequent degradation of inhibitors of kappa B (IκBs) trigger NF-κB activation primarily through the activation of the IκB kinase (IKK) [15]. The MAPK pathway can stimulate the translocation of NF-κB from the cytoplasm to the nuclei and its p65-mediated transactivation of proinflammatory mediators [16]. It has been reported that the MAPK families include extracellular signal-regulated kinase (ERK), c-Jun N-terminal kinase (JNK), and p38 kinase. Therefore, targeting the MAPK pathway is a significant and attractive therapeutic anti-inflammatory method [17].

This study aims to identify apigetrin through NF-κB and MAPK signaling pathways and evaluate biological activity in the presence of different apigetrin concentrations along with the presence of LPS. The anti-inflammatory activity of apigetrin and the mechanism by which it modulates the inflammatory response are investigated.

## 2. Materials and Methods

### 2.1. Reagents and Chemicals

Apigetrin was purchased from InterPharm (purity: ≥98%, Koyang-si, Korea). Rat skeletal muscle cell line L6 was obtained from the Korean Cell Line Bank (Seoul, Korea). Dulbecco’s modified Eagle’s medium (DMEM), fetal bovine serum (FBS), phosphate-buffered saline (PBS), and antibiotics penicillin/streptomycin (P/S) were purchased from Gibco (BRL Life Technologies, Grand Island, NY, USA). 3-(4,5-dimethylthiazol-2-yl)-2,5-diphenyltetrazolium bromide (MTT) was purchased from Duchefa Biochemie (Haarlem, Netherlands). Antibodies of iNOS (CST, Cat. No. 13120S), COX-2 (CST, Cat. No. 12282S), phospho-p65 (CST, Cat. No. 3033S), p65 (CST, Cat. No. 8242S), phospho-IκB-α (CST, Cat. No. 2859S), IκB-α (CST, Cat. No. 4812S), phospho-JNK (CST, Cat. No. 4671S), JNK (CST, Cat. No. 9285S), phospho-p38 (CST, Cat. No. 9216S), p38 (CST, Cat. No. 8690S), phospho-ERK (CST, Cat. No. 4370S), ERK (CST, Cat. No. 4695S), and β-actin (CST, Cat. No. 4970S) were purchased from Cell Signaling Technology (Danvers, MA, USA). Horseradish peroxidase (HRP)-conjugated secondary antibodies to antirabbit (Bethyl, Cat. No. A120-101P) and antimouse (Bethyl, Cat. No. A90-116P) were obtained from Bethyl Laboratories, Inc. (Montgomery, AL, USA).

### 2.2. Cell Culture and Apigetrin Treatment

The L6 skeletal muscle cells were cultured in complete DMEM containing 10% FBS and supplemented with 100 U/mL penicillin and 100 μg/mL streptomycin (P/S). The cells were incubated at 37 °C in a humidified atmosphere containing 5% CO_2_. The cells were treated with the indicated concentrations of apigetrin with or without lipopolysaccharide (LPS) (2 μg/mL) for 24 h in a complete medium. Concentrations were selected depending upon low cytotoxicity from MTT assay [4].

### 2.3. Cell Viability Assay

Cell viability was analyzed using 3-(4,5-dimethylthiazol-2-yl)-2,5-diphenyltetrazolium bromide (MTT). The L6 cells were seeded into 48-well plates at 3 × 10^4^ cells per well and grown for 18 h. After treatment with the indicated concentrations of apigetrin (0, 1, 2, 5, 10, and 15 μM), with or without LPS (2 μg/mL), the cells were incubated for 24 h. The 0.5% MTT solution was added to each well, and the cells were incubated for 2 h at 37 °C in the incubator. The insoluble formazan was solubilized in dimethylsulfoxide (DMSO), and then absorbance was measured at 540 nm with PowerWave HT microplate spectrophotometry (BioTek, Winooski, VT, USA).

### 2.4. Western Blotting

The L6 cells were seeded into 60 mm plates at 4 × 10^5^ cells per well and treated with the indicated concentrations of apigetrin (0, 5, and 10 μM), with or without LPS (2 μg/mL), for 24 h. Then, the incubated cells were lysed using radioimmunoprecipitation assay (RIPA) buffer (iNtRON Biotechnology, Gyeonggi, Korea) containing a protease inhibitor cocktail and a phosphatase inhibitor (Thermo Fisher Scientific, Waltham, MA, USA). The protein quantification of each cell lysate sample was measured using bicinchoninic acid (BCA) assay (Thermo Fisher Scientific, Waltham, MA, USA) according to the manufacturer’s instructions. Equal volumes of protein (~10 μg) were separated on 8–15% sodium dodecyl sulfate (SDS)-polyacrylamide gels and then transferred to a polyvinylidene fluoride (PVDF) membrane (Atto Co., Ltd., Tokyo, Japan) using a semi-dry transfer system (Atto Corp., Tokyo, Japan). The membranes were blocked with 5% bovine serum albumin (BSA) in tris-buffered saline containing 1% Tween 20 (TBS-T, pH7.4) at room temperature (RT) for 1 h, followed by incubation overnight at 4 °C with a 1:1000 dilution of the respective primary antibody. The membranes were washed with TBS-T buffer every 10 min in five repetitions at RT and then incubated with horseradish peroxidase (HRP)-conjugated secondary antibody for 2 h at RT. The membranes were then rewashed using TBS-T, detected with electrochemiluminescence (ECL) reagent (Bio-Rad, Hercules, CA, USA), and analyzed using Image Lab 4.1 (Bio-Rad) program. The densitometry analysis was performed using Image J software (U.S. National Institutes of Health, Bethesda, MD, USA).

### 2.5. Molecular Docking Analysis

To perform molecular docking analysis, the protein structure of NF-κB from PDB (https://www.rcsb.org, accessed on 20 May 2022) was retrieved (protein ID: 4Q3J), and the compound structure of apigetrin was downloaded from PubChem (compound ID: 5280704) (https://pubchem.ncbi.nlm.nih.gov/, accessed on 20 May 2022). We performed docking analysis using USCF chimera (https://www.cgl.ucsf.edu/chimera/, accessed on 20 May 2022) with the default parameters. The affinity of the binding was determined using estimated free energy binding and total intermolecular energy.

### 2.6. Statistical Analysis

All experimental results were expressed as the mean ± standard deviation (SD) of triplicate samples. Significant differences between groups were calculated by one-way factorial analysis of variance (ANOVA) followed by a Tukey’s post hoc test, and *p* < 0.05 was considered statistically significant. ^#^ *p* < 0.05, ^##^ *p* < 0.01, ^###^ *p* < 0.001 vs. untreated group; and * *p* < 0.05, ** *p* < 0.01, *** *p* < 0.001 vs. LPS-alone-treated group.

## 3. Results

### 3.1. Effects of Cytotoxicity by Apigetrin on L6 Cells

**Figure 1:**
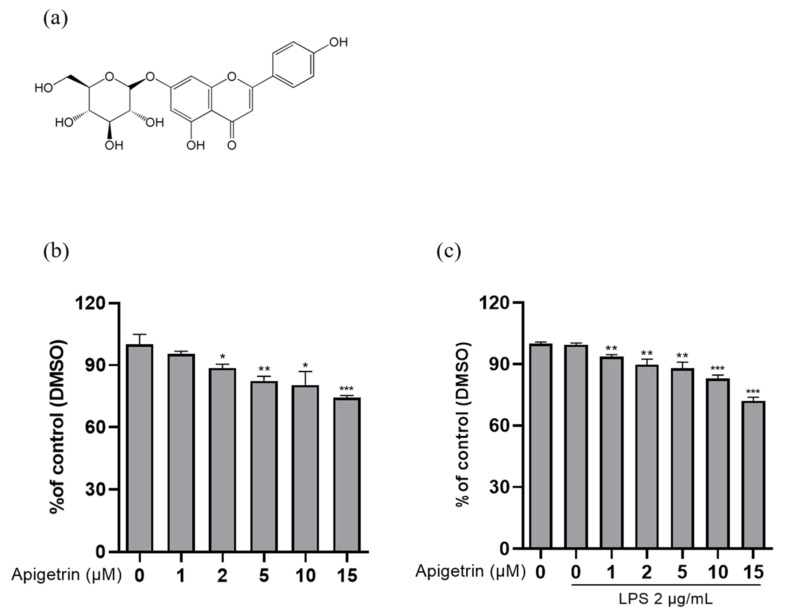
Cytotoxic effect of apigetrin in L6 cells. (**a**) The chemical structure of apigetrin. (**b**) L6 cells were treated with apigetrin (0, 1, 2, 5, 10, and 15 μM) for 24 h. (**c**) L6 cells were treated with apigetrin (0, 1, 2, 5, 10, and 15 μM), with or without LPS (2 μg/mL), for 24 h. The results are presented as the mean ± standard deviation (SD) of three independent experiments. * *p* < 0.05; ** *p* < 0.01; *** *p* < 0.001 vs. untreated group and the LPS-alone-treated group, respectively.

To identify the cytotoxicity of apigetrin, L6 cells were treated with various concentrations of apigetrin (0, 1, 2, 5, 10, and 15 μM) for 24 h (Figure 1b). Further, we performed an MTT assay cotreated with apigetrin and LPS 2 μg/mL. With the cotreated groups, we chose 5 and 10 μM concentrations, which showed, approximately, up to 20% inhibition in L6 cells (Figure 1c). Therefore, doses of 5 and 10 μM were used in subsequent experiments.

### 3.2. Observation of Morphological Changes on LPS and Apigetrin-Treated L6 Cells

**Figure 2:**
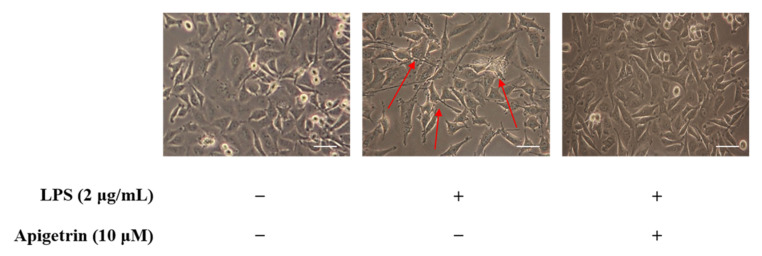
Morphological changes of L6 cells. Changes were observed after 24 h of treatment with apigetrin (0 and 10 μM), with or without LPS (2 μg/mL), at indicated concentrations. The observations were carried out with the aid of a light phase-contrast microscope (×200).

In this study, we investigated whether apigetrin could recover LPS-induced inflammation in L6 cells using skeletal muscle cells. Apigetrin (0 and 10 μM) was treated with or without LPS (2 μg/mL) for 24 h. In the area indicated by the arrows, swelling and pseudopodium formation induced by LPS were observed in the morphology of LPS-alone-treated group cells. Treatment with LPS and apigetrin cells, on the other hand, demonstrated no significant change when compared to the untreated LPS control group (Figure 2).

### 3.3. Apigetrin Suppressed LPS-Induced iNOS and COX-2 Proteins Expression

**Figure 3:**
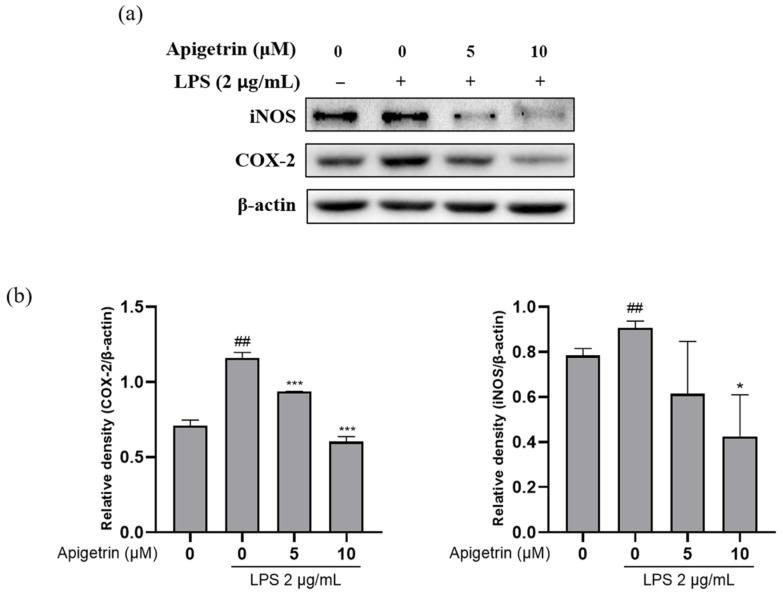
(**a**) Effect of apigetrin on the expression of iNOS and COX-2. L6 cells were treated with apigetrin (0, 5, and 10 μM), with or without LPS (2 μg/mL), for 24 h. Protein expression was determined by Western blotting. (**b**) The results are presented as the mean ± standard deviation (SD) of three independent experiments. ^##^ *p* < 0.01 vs. untreated group; * *p* < 0.05 vs. LPS-alone-treated group; *** *p* < 0.001 vs. LPS-alone-treated group.

The effect of apigetrin on inducible nitric oxide synthase (iNOS) and cyclooxygenase-2 (COX-2) expression levels in LPS-induced L6 cells was investigated with Western blotting. The L6 cells treated with LPS (2 μg/mL) alone significantly increased compared to the untreated LPS control group at the iNOS and COX-2 expression levels. However, the expression levels of iNOS and COX-2 in the L6 cells significantly decreased in a dose-dependent manner when cotreated with apigetrin (0, 5, and 10 μM) for 24 h (Figure 3a,b). The results showed that apigetrin suppressed LPS-induced iNOS and COX-2 expression levels in L6 cells.

### 3.4. Effects of Apigetrin on NF-κB Signaling in LPS-Treated L6 Cells

**Figure 4:**
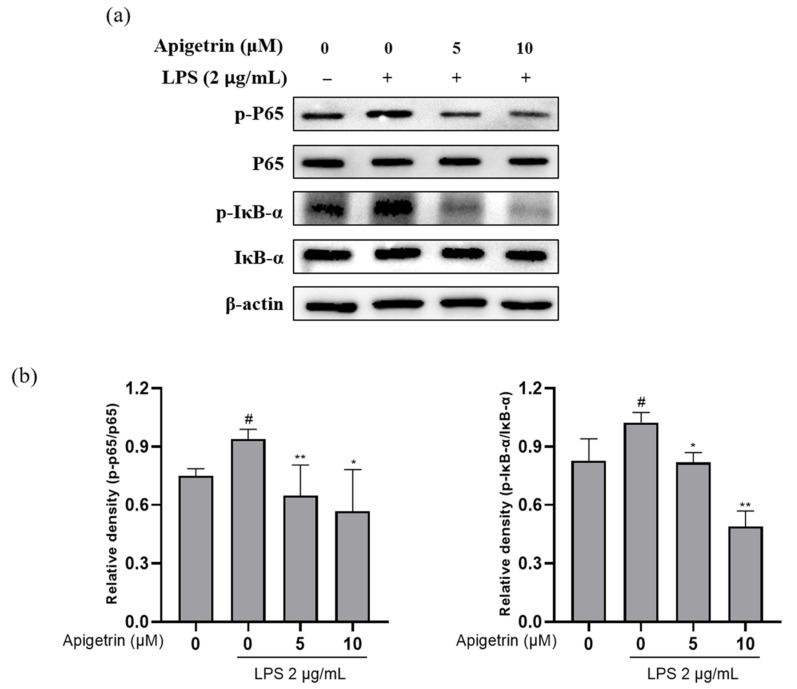
(**a**) Inhibitory effect of apigetrin on the LPS-induced protein expression of NF-κB signaling in L6 cells. L6 cells were treated with apigetrin (0, 5, and 10 μM), with or without LPS (2 μg/mL), for 24 h. Protein expression was determined by Western blotting. (**b**) The results are presented as the mean ± standard deviation (SD) of three independent experiments. ^#^ *p* < 0.05 vs. untreated group; * *p* < 0.05 vs. LPS-alone-treated group; ** *p* < 0.01 vs. LPS-alone-treated group.

The effect of apigetrin on the LPS-induced phosphorylation of nuclear factor-kappa B (NF-κB) proteins and the expression levels of the inhibitor of IκB-α and p65 were examined with Western blotting. The results indicated that the apigetrin (0, 5, and 10 μM) treatment following LPS (2 μg/mL) stimulation decreased p-IκB-α and p-p65 protein expression, whereas the expression of p65 and IκB-α remained unchanged (Figure 4a,b). Based on these results, we suggested that apigetrin decreased protein levels by downregulating the phosphorylation of p65 and IκB-α in L6 cells.

### 3.5. Effects of Apigetrin on MAPK Phosphorylation in LPS-Treated L6 Cells

**Figure 5:**
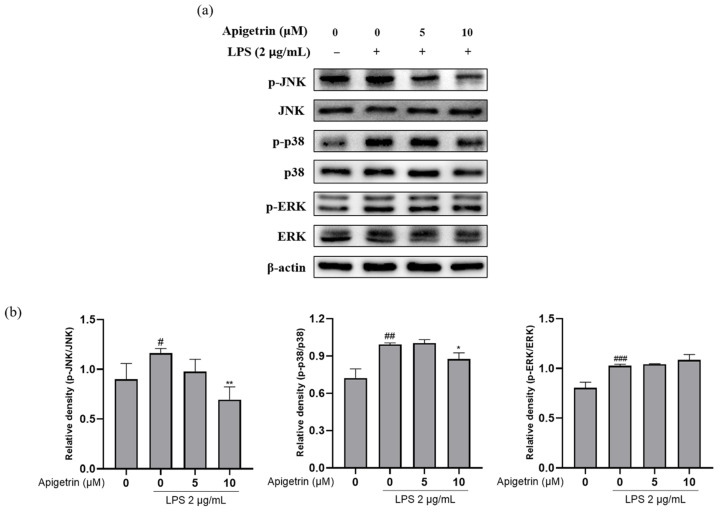
(**a**) Inhibitory effect of apigetrin on the LPS-induced protein expression of MAPK phosphorylation in L6 cells. L6 cells were treated with apigetrin (0, 5, and 10 μM), with or without LPS, (2 μg/mL) for 24 h. Protein expression was determined by Western blotting. (**b**) The results are presented as the mean ± standard deviation (SD) of three independent experiments. ^#^ *p* < 0.05 vs. untreated group; ^##^ *p* < 0.01 vs. untreated group; ^###^ *p* < 0.001 vs. untreated group; * *p* < 0.05 vs. LPS-alone-treated group; ** *p* < 0.01 vs. LPS-alone-treated group.

The level of phosphorylated mitogen-activated protein kinases (MAPKs) was evaluated to investigate how apigetrin affected MAPK, which was activated by LPS. The phosphorylation of c-Jun N-terminal kinase (JNK), p38, and extracellular signal-regulated kinase (ERK) was observed following treatment with LPS (2 μg/mL) alone. When cotreated with apigetrin (0, 5, and 10 μM), the expression levels of p-JNK and p-p38 were downregulated in phosphorylation levels compared to the LPS-alone treated group. However, apigetrin did not affect ERK phosphorylation (Figure 5a,b). Our findings indicated that apigetrin inhibited the phosphorylation of JNK and p38 more significantly than it inhibited the phosphorylation of ERK in L6 cells.

### 3.6. Molecular Docking with Apigetrin and NF-κB

**Figure 6:**
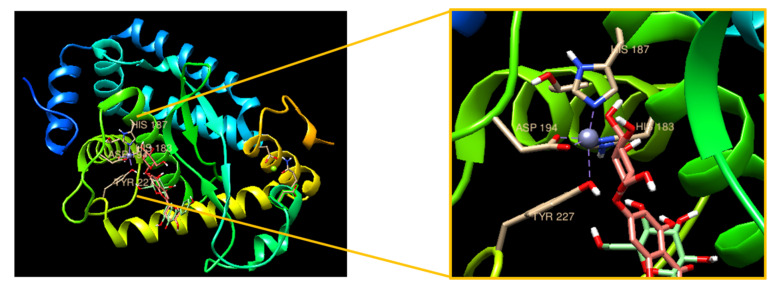
In silico molecular docking analysis of the ligand apigetrin with target NF-κB. The 3D structure of NF-κB bound efficiently with the compound apigetrin with its interacting amino acids TYR, ASP, and HIS.

Additionally, we performed an in silico molecular docking analysis to confirm the interaction of apigetrin with NF-κB. Figure 6 shows the bound complex of apigetrin with NF-κB to ligand-protein docking using UCSF Chimera software. The molecular dock score revealed a binding affinity with an estimated free energy of −8.5 kcal/mol by NF-κB, followed by high free energy of apigetrin. The interacting amino acid residues involved in the bound complex were TYR 227, ASP 194, HIS 183, and HIS 187. Overall, our findings support that apigetrin has an anti-inflammatory effect by blocking NF-κB signaling.

## 4. Discussion

Apigetrin belongs to the flavonoid glycoside class and can be isolated from herbal plants, such as *Scutellaria Baicalensis georgi*, *Teucrium gnaphalodes*, *Matricaria chamomilla*, and *Stachys tibetica vatke* [4]. Accordingly, apigetrin has been shown to have neuroprotective properties by inhibiting inflammation and oxidative stress [18]. Additionally, anti-inflammatory effects have been reported in the aglycone apigenin in different types of skeletal muscle cells [19,20]. However, the effects of apigetrin on anti-inflammation in L6 cells have not been fully reported. In the present study, we demonstrated the anti-inflammatory effects of apigetrin using the skeletal muscle cell line L6.

Apigetrin showed low cytotoxicity against L6 cells, with or without lipopolysaccharide (LPS) treatment. In pathological conditions, key inflammatory mediators, inducible nitric oxide synthase (iNOS) and cyclooxygenase-2 (COX-2), aggravated inflammation and the inflammatory response by interacting synergistically with proinflammatory cytokines [21,22]. Next, the inhibitory effect of apigetrin on iNOS and COX-2 was examined. Our results showed that treatment with apigetrin affected the activity of the cytokines.

The ubiquitination and proteasome degradation of IκB activates dimeric nuclear factor-kappa B (NF-κB), causing it to translocate from the cytoplasm to the nucleus where it can transcribe target genes [23,24]. In addition, it has been reported that, following the proteolytic degradation of NF-κB-binding, IκB-α kinase proteins, such as IκB-α and p65, promote the response of cytokine genes [25]. The NF-κB signaling dysregulation inhibits muscle growth and regeneration, as well as perpetuates inflammation in muscle diseases [26]. Our results showed that apigetrin significantly suppressed NF-κB signaling activation through the inhibition of p65 and IκB-α phosphorylation by LPS stimulation. Similarly, the previous documented study confirmed that apigetrin induces an anti-inflammatory effect through the NF-κB pathway in human middle ear epithelial cells (HMEECs) [4]. In addition, the molecular docking analysis showed that, as a result, SCU can bind with NF-κB and it may inhibit the NF-κB pathway. This suggests that SCU has an anti-inflammatory effect on macrophage cells.

Mitogen-activated protein kinases (MAPKs) are known to play a role in NF-κB activation [27]. During inflammatory processes, LPS may activate MAPK, including at least three families of c-Jun N-terminal kinase (JNK), p38, and extracellular signal-regulated kinase (ERK), which, subsequently, phosphorylate and activate transcription factors [28]. In addition, MAPK activation is a metric that measures how much mechanical stress is applied to the muscle [29]. The effects of apigetrin on JNK, p38, and ERK phosphorylation levels were investigated. Apigetrin significantly inhibited the phosphorylation of JNK and p38, but did not affect the ERK expression as similarly reported by Cui, Yijun et al. [30]. Therefore, it can be stated that the present study supports that apigetrin inhibits the translocation of NF-κB and MAPK signaling pathways in L6 skeletal muscle cells.

## 5. Conclusions

**Figure 7:**
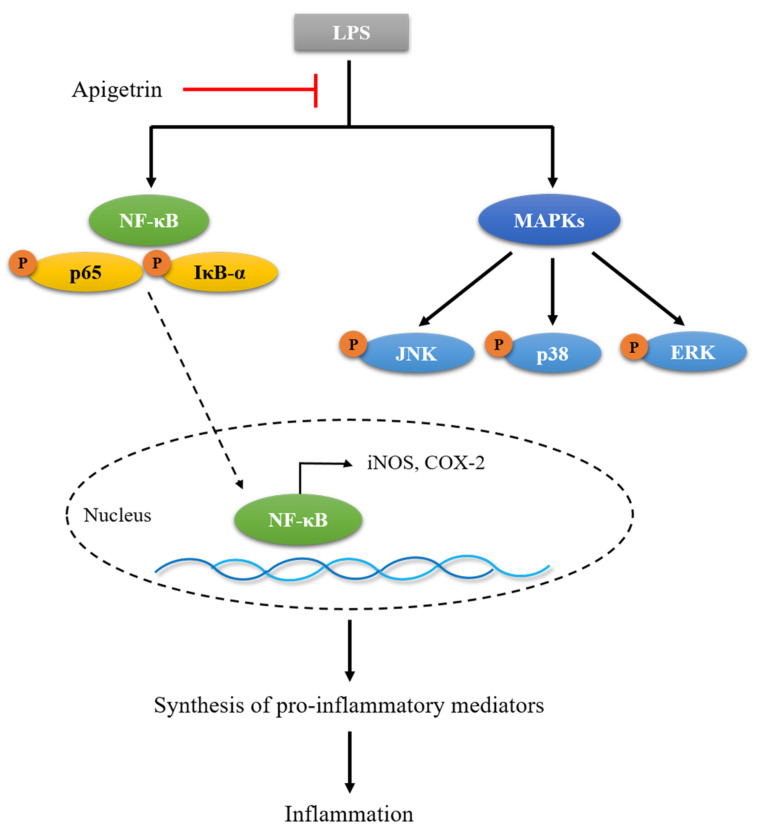
The proinflammatory pathway of apigetrin in L6 cells.

In conclusion, the mechanisms regulating the anti-inflammatory activity of apigetrin were not revealed completely in the present study. In our study, we demonstrated that apigetrin inhibited inflammatory mediators in L6 skeletal muscle cells. According to these findings, apigetrin possesses anti-inflammatory properties and may act as a modulator of skeletal muscle inflammation processes (Figure 7). However, our study had limitations, such as only using the L6 skeletal muscle cell line. Therefore, we plan to adopt an experimental module in other cell lines.

## Data Availability

Not applicable.
